# The Big Man Mechanism: how prestige fosters cooperation and creates prosocial leaders

**DOI:** 10.1098/rstb.2015.0013

**Published:** 2015-12-05

**Authors:** Joseph Henrich, Maciej Chudek, Robert Boyd

**Affiliations:** 1 Department of Human Evolutionary Biology, Harvard University, 11 Divinity Avenue, Cambridge, MA 03138, USA; 2 Department of Psychology, University of British Columbia, 2136 West Mall, Vancouver, British Columbia, Canada V6S 1V9; 3 Department of Economics, University of British Columbia, 2136 West Mall, Vancouver, British Columbia, Canada V6S 1V9; 4 Canadian Institute for Advanced Research, 180 Dundas Street, Toronto, Ontario, Canada M5G 1Z8; 5 Institute of Human Origins, School of Human Evolution and Social Change, Arizona State University, Tempe, AZ 85281, USA

**Keywords:** prestige, status, cooperation, prosociality, prestige-biased transmission, cultural evolution

## Abstract

Anthropological evidence from diverse societies suggests that prestige-based leadership may provide a foundation for cooperation in many contexts. Here, inspired by such ethnographic observations and building on a foundation of existing research on the evolution of prestige, we develop a set of formal models to explore when an evolved prestige psychology might drive the cultural evolution of *n*-person cooperation, and how such a cultural evolutionary process might create novel selection pressures for genes that make prestigious individuals more prosocial. Our results reveal (i) how prestige can foster the cultural emergence of cooperation by generating correlated behavioural phenotypes, both between leaders and followers, and among followers; (ii) why, in the wake of cultural evolution, natural selection favours genes that make prestigious leaders more prosocial, but only when groups are relatively small; and (iii), why the effectiveness of status differences in generating cooperation in large groups depends on cultural transmission (and not primarily on deference or coercion). Our theoretical framework, and the specific predictions made by these models, sketch out an interdisciplinary research programme that cross-cuts anthropology, biology, psychology and economics. Some of our predictions find support from laboratory work in behavioural economics and are consistent with several real-world patterns.

## 1. Introduction

Ethnography suggests that particularly prestigious individuals generate cooperation, influence group decisions and provide informal leadership in a wide range of societies [1]. These individuals often derive their prestige from superior skill, knowledge or success in locally valued domains, including domains related to social norms and rituals. In small-scale societies, the domains associated with prestige include hunting, oratory, shamanic knowledge and combat. Prestigious individuals, particularly those who emerge as local leaders, often behave prosocially and exhibit unusual levels of generosity. They also receive deference from others in many forms including public praise, small gifts, ready aid in projects, a variety of favours and mating opportunities [1–4]. Though these patterns can be observed across a wide range of human societies, they are particularly important in egalitarian or acephalous tribal societies, including mobile hunter–gatherers, which have relatively few institutions for selecting leaders, granting authority or delivering sanctions. Among the Andaman Islanders, for example, Radcliffe-Brown writes ([5, p. 45]; italics are ours):

Besides the *respect for seniority*, there is another important factor in the regulation of social life, namely the respect for certain personal qualities. These qualities are *skill in hunting and warfare, generosity and kindness, and freedom from bad* temper. A man possessing them inevitably acquires a position of *influence* in the community. His *opinion on any subject carries more weight* than that of another even older man. The *younger men attach themselves to him*, are anxious to please him *by giving him any presents that they can, or by helping him in such work* as cutting a canoe, and to join him in hunting parties or turtle expeditions … In each local group there was usually to be found *one man who thus by his influence could control and direct others*.

Radcliffe-Brown identified a cluster of traits that seemed to give some individuals—usually one in each group—particular influence in their communities. These traits, including both skill or knowledge in a locally valued domain and an unusual level of prosociality, attracted less prestigious individuals who willingly paid deference to this high status person, and seemed to desire just to hang around them. When the prestigious individual went hunting or to make a canoe, this crew of subordinates voluntarily went along to help.

Such prestige-based status hierarchies and patterns of informal leadership are common in egalitarian societies [1,6]. For example, summarizing work on hunting, status and cooperation among foraging peoples in Northern Canada, Paine writes, ‘Acknowledged expertise attracts, though perhaps only temporarily, what we may term a following of dependent persons. These persons will be welcomed as a principal source of prestige—as a capital benefit of the hunter's expertise’ [7, p. 165]. Similarly, in the Kalahari Desert in southern Africa, Lee [8, pp. 343–344] describes the subtle patterns of informal leadership, explaining that skilled hunters, ritual specialists, orators or arguers ‘may speak out more than others, may be deferred to by other discussants' in group discussions, and that ‘their opinions hold a bit more weight’. In the same vein, Marshall [9] observed that particularly skilled Kalahari hunters, well recognized for their success, act as informal leaders for hunting parties (also see [10]; [11, p. 155]).

It is particularly noteworthy that prestige status shapes social life and provides a foundation for informal leadership in groups possessing a variety of social norms and practices that otherwise actively suppress status differences [12], and where any substantial accumulation of material wealth is impossible. In the Kalahari, for example, individuals that begin to accumulate more than a couple of hunting successes in a row take time off to avoid the envy of others [13, p. 53]. Credit for hunting success is further diffused by sharing arrowheads, and assigning the ownership of a kill to the owner of the arrowhead instead of the hunter. And, famously, the hunter's band actively ‘insults' the quality of his kills to deflate his pride and ‘cool his heart’ [8, p. 246].

In sedentary societies that lack institutions for transmitting power across generations, prestigious ‘Big Men’ emerge and often become the centre of political life. As in more mobile populations, prestige is often derived from skill, expertise and success in locally valued domains, but now these domains include economic production or wealth accumulation. Such societies can be found all over the world [13], including among foragers in California and the Northwest Coast of North America [14,15]. However, this syndrome has been particularly well documented in Melanesia, where it sometimes takes quite elaborate forms [16,17] called the ‘Big Man Complex’ [17,18].

In a classic paper, Sahlins [19] describes the leadership of the Melanesian Big Man as the ‘outcome of a series of acts which elevate a person above the common herd and attract about him a coterie of loyal, lesser men.’. The local terms for ‘Big Man’ are informative, translating variously as ‘man of renown’, ‘generous rich-man’, ‘centre-man’ and, of course, ‘big-man’. Leadership here depends not on institutional roles, but entirely on an individual's ability to generate followership. The foundation of a Big Man's influence derives from his demonstration of skills that command respect, including gardening, oration, bravery in war and magic, and an ability to successfully deploy these skills in substantial cooperative endeavours [16]. Within a Big Man's primary sphere of influence, which rarely exceeds 80 individuals [19], people follow his lead, allowing him to effectively organize economic production. The proceeds from these endeavours can then be given away, to create debts and reciprocal obligations that further expand his influence. Successes attract more loyal followers, expanding the Big Man's faction and further elevating his prestige.

The connection between skill/knowledge, prestige, deference, attracting followers and cooperation has been observed throughout Melanesia, including in the so-called ‘Great Man’ societies [16–18]. However, in societies with the elaborated ‘Big Man Complex’, the opportunities created by more generalized currencies of exchange (like pigs, shell monies and yams) create a niche for self-aggrandizing Big Men and their factions to use complex webs of debts, reciprocal obligations and alliances to compete with other Big Men, and their factions. Thus, the Melanesian ‘Big Man Complex’ goes well beyond the informal prestigious leaders found in many societies. However, as Sahlins emphasizes, the crucial core of this process is not the high-level strategic manipulation of the Big Man, but his initial ability to attract and motivate a constellation of followers.

Several of these patterns of prestige-based leadership can be accounted for by a culture–gene coevolutionary approach. Based on ideas developed by Henrich & Gil-White [4] and Boyd & Richerson [4,20], this body of theory proposes that the emergence of cultural learning unleashed a culture–gene coevolutionary interaction that in turn created a form of status in humans, *prestige*, that is distinct from the *dominance* status seen in other social mammals—status based on strength, intimidation, and the ability and willingness of some individuals to impose their will on others. Existing work explains why people are attracted to, and deferential towards, individuals who are particularly successful, skilled or knowledgeable in locally valued domains. It also explains why prestigious individuals tend to be particularly persuasive, why their opinions carry more weight than others even on topics well-outside of their expertise, and why they are disinclined towards coercive tactics and personal antagonisms with others (unlike dominant individuals). Finally, it explains how prestige assessments—based on the deferential and imitative cues inadvertently given off by learners—can develop separately from direct evaluations of a person's expertise, success or skill (more on this later). Thus, this work provides a plausible theoretical account for (i) the tendency of particularly skilled or successful individuals to attract a following, (ii) the tendency of followers to both imitate and pay deference to prestigious individuals, and (iii) the patterns of influence and persuasion that prestigious individuals create across a broad range of behavioural domains.

In the following, we explore two primary questions that flow from this work.

(1) If, as the theory predicts, lower status individuals in a social group are inclined to copy particularly prestigious individuals, then prestigious individuals who are more cooperative might be able to induce greater cooperation among their followers. When can such a *prestige effect* induce the spread of cooperative cultural traits over generations?
(2) If prestigious individuals can induce enough cooperativeness via their own actions and the imitation of those actions by others, then natural selection may favour genes that make prestigious individuals more cooperative because, by being more cooperative themselves, they create a more cooperative environment. Under what conditions will such ‘cooperative genes' spread?

Here, we are focused on *prestige* as it derives from the informational goods first made available with the evolution of cultural learning in humans. Of course, individuals may derive forms of prestige by possessing other means to bestow benefits on others, such as by having large social networks of friends, allies or suitors that others could tap. However, explaining the full breadth of psychological (e.g. unconscious mimicry), ethological (e.g. proximity maintenance) and sociological (network structure) patterns of prestige requires a central role for informational goods [4,21]. For example, non-informational approaches to prestige cannot explain why learners, from a young age, use prestige cues to bias their imitation [22,23] or why particularly skilled athletes are sought out for advice in a wide range of domains [24]. Moreover, as we demonstrate formally below, when cultural learning is important, the information-goods form of prestige can provide a particularly potent mechanism to generate both cooperation in followers and generosity in high status individuals. However, when followers merely go along out of deference to high status individuals, little cooperation or generosity is generated in our model.

By creating a voluntary coterie of followers keenly tuned into their leader, such prestige-based leadership can lay a foundation to support other non-informational forms of status. A following provides a network of like-minded allies that can support non-informational forms of prestige, for example by creating valuable social connections. Or, the collective action potential created by prestige-based leadership can provide coercive threats—that is, dominance (see below). Thus, an individual with information-based prestige, through his ability to generate collective action, can augment his influence through both dominance and non-informational prestige, as well as other mechanisms such as reciprocity. The Big Man's core following, for example, may be those he attracts via his informational prestige (along with his kin). He then deploys this coterie to increase his status and influence. The result is multifaceted leadership: he possesses informational prestige (and kinship) towards his core, non-informational prestige towards a near periphery who are not attracted by his knowledge or skill but do recognize his capacity to generate non-informational benefits (often via collective action), and an outer periphery who are compelled into compliance via coercive threat.

Prestige-based leadership may provide a foundation for the emergence of more formal, enduring systems for selecting leaders (e.g. blood lines, elite councils or democratic elections). However, even in complex societies, prestige and prestige-based leadership play a central role: political succession, for example, can depend on sons' individual merits [25]; and when these hereditary chiefs are challenged, it is often by a prestigious military commander [26]. Even in modern organizations, where power is formalized, a leader's effectiveness often seems to depend on his or her prestige. Of course, prestige-based leadership continues to play an important role in sports teams [24], informal working groups [27], political parties, emergency rooms, schoolyard cliques and academic departments.

Our work complements existing lines of research that explore how individual differences (e.g. in fighting ability or allies) combine with mechanisms based on signalling [28], punishment [29–31] and reputation [1,32] to explain the relationship among leadership, cooperation and generosity.^1^ Here, we deliberately put aside these standard evolutionary approaches—applicable to many species—to focus on a novel mechanism at the nexus of status, leadership and cooperation, which we argue arose in humans via culture–gene coevolution. The goal is to see how much cooperation in followers and generosity in leaders it can generate without building in punishment, repetition, reputation, signalling or individual asymmetries (except for informational asymmetries). Note, unlike some approaches that focus on how leadership can improve coordination [36], we have focused on *n*-person cooperative dilemmas because these best capture the real-world situations we want to explain, such as feasting, barbasco fishing, raiding, rabbit hunting, community defence, house construction, etc. In the following, we first sketch the theoretical background for our approach, and then develop a series of models to address our two key questions.

## 2. Theoretical background

Humans are a cultural species, entirely dependent on learning vast repertoires of techniques, skills, motivations, norms, languages and know-how from others in their social groups [21,35]. To understand this unique feature of our species, researchers have focused on understanding how natural selection might have given rise to our evolved capacities to learn from others—cultural learning—and how the emergence of this capacity subsequently gave rise to a second system of inheritance—cultural evolution—that has long interacted with, and at times driven, our genetic evolution [20,37]. Supporting this broad view, many lines of evidence increasingly suggest that culture–gene coevolutionary interactions are crucial for understanding human anatomy, physiology and psychology [21,38].

### (a) The evolution of prestige

Operating within this framework, Henrich & Gil-White [4] proposed an evolutionary approach to human status (also see [21, ch. 8]). They argue that a second form of status emerged in humans in response to the new informational dynamics generated by cumulative cultural evolution. As noted, this second form of status—*prestige*—emerged alongside a phylogenetically older form of status—*dominance*—that we share with many other species. Individuals are granted prestige when others perceive them to possess valuable skills and knowledge in locally valued domains. Aspiring learners pay deference to these individuals in return for more learning opportunities. By contrast, deference is granted to dominant individuals to the degree that others perceive them as willing and able to use physical force or other coercive tactics if deference is not paid. Each type of status is associated with a particular suite of strategies, emotions, motivations and ethological displays, and each results in distinct sociological patterns [21,24,39].

On this account, the evolution of prestige can best be understood in three major evolutionary steps:

(i) *Model-ranking in cultural learning*. As the social learning abilities of our ancestors increased, learners could acquire details of behaviour from those they were learning from—their *models*. This created a selection pressure to be careful in choosing models, which in turn drove the evolution of both the abilities and motivations to use cues to rank potential models according to who is most likely to possess fitness-enhancing skills and know-how.
(ii) *Prestige deference*. The evolution of model-ranking abilities created competition among learners for access to the most highly ranked models. Such competition then favoured the evolution of motivations to deliver benefits—freely conferred deference—to the most highly ranked models in exchange for informational access—for learning opportunities and teaching. Prestige deference could come in many forms, including (i) help with their projects, (ii) deference in conversations, (iii) public praise and verbal support, and (iv) gifts.
(iii) *Prestige-biased cultural learning*. The emergence of model-ranking capacities, the ensuing competition among learners for access to the best models, and the differential bestowal of benefits on the most highly ranked would have generated distinct patterns, and thereby another evolutionary opportunity. By attending to who other learners are watching, listening to, deferring to and imitating, learners can improve their own model-rankings. Particularly when learners are inexperienced or poorly equipped to evaluate highly skilled performances, or when it is difficult to accurately differentiate skills, knowledge and success, following the inadvertent ‘prestige cues'—attention, deference and mimicry—given off by other learners allows individuals to augment their own model-ranking assessments and more accurately identify the best models to learn from. This is a second-order form of cultural learning in which learners can infer who other learners think are worthy of learning from.

This approach predicts that learners use cues of success, skill and prestige—among others—to figure out who to learn from. However, such cues do not tell learners what aspects of their model's behaviour or traits are causally linked to their model's success or skill. For many traits, the causal linkages to the model's success will be cognitively opaque or simply too costly to figure out. Consequently, the theory predicts that learners will tend to copy their preferred models broadly, and in ‘bundles'. This means they will often copy many traits that turn out not to be causally connected at all with their models' success, skill or competence. To see this, consider a young learner who is watching the best hunter in her community, with the aspiration of someday being a great hunter herself. Should our learner copy her model's practices of (i) departing early in the morning, (ii) eating a lot of carrots, (iii) saying a quick prayer prior to releasing his arrow, (iv) putting charcoal on his face, and (v) adding a third feather to his arrow's fletching? Any or all of these may contribute to the hunter's success. But our learner just cannot tell, so she copies most or all of these. Of course, some aspects of a model's behaviour may seem obviously connected to a models' success or competence, so these may be copied more readily. But the products of cumulative cultural evolution possess crucial adaptive complexity that practitioners themselves do not comprehend, so strategies that restrict learners to only copying causally well-understood elements are evolutionary losers [21,38].

This theory, then, provides an explanation for many of the ethnographic patterns observed above. Highly skilled or knowledgeable individuals attract many followers because they are perceived to possess valuable cultural know-how, which learners can acquire if they hang around. Such individuals receive deference because learners need to pay prestigious individuals for access, for learning opportunities. Skill, success and expertise turn into prestige, as learners alter their views of others in response to the patterns of attention, deference and imitation they observe. Finally, prestigious individuals become highly influential and naturally persuasive both because others are broadly inclined to selectively learn from them over others (biased cultural learning in bundles) and as a means of paying deference.

### (b) Empirical evidence

Many predictions have been derived from this theory and tested in various ways, both in the laboratory and in the field ([4]; [21, ch. 8]). For example, psychological research using university sports teams shows that prestige and dominance form two distinct and uncorrelated status hierarchies with different emotional and personality profiles [24]. Paralleling Radcliffe-Brown's observations, prestigious individuals—in contrast to dominant individuals—tended to be kind, free from bad temper and sought out for advice on many topics. Complementing this fieldwork, laboratory studies also reveal distinct prestige-based and dominance-based strategies for attaining influence (informal leadership) in small, ‘minimal’, groups, with each type of status characterized by distinct vocal patterns, ethological displays, emotions [27] and hormonal signatures [40]. Finally, anthropological research among the Tsimane' in the Bolivian Amazon reveals that both prestige and dominance are associated with higher fitness, though this is achieved via somewhat different routes [3,41].

For our purposes here, there are three crucial empirical questions

(i) Do individuals use cues of success, competence, skill, knowledge and prestige in figuring out who to learn from?
(ii) Does this apply to a wide range of behaviours, traits or motivations, including those not obviously connected to the individual's expertise or source of prestige?
(iii) Do learners use cultural learning to acquire costly social behaviour and motivations, including those related to cooperation?

Much evidence suggests that the answers to all three questions are yes. To the first question, several lines of empirical work confirm that individuals do use cues of success, competence, skill, knowledge and prestige in figuring out who to learn from. In the laboratory, this is well established in infants [42,43], children (see reviews in [44,45]) and adults [4,46] across a range of domains. In the field, the construction of cultural transmission networks on Yasawa Island, Fiji [47] shows that individuals aggregate a wide range of cues to better target their cultural learning, including cues related to success, knowledge and age.

On the second question, evidence also indicates that individuals use cues of success and skill across many domains (e.g. acting skill influences the transmission of medical decisions). In the laboratory, young children reveal cross-domain effects when they use a model's accuracy in the domain of object labelling as a cue in copying what the model does with novel artefacts [48]. Similar research shows that ‘prestige cues', which involve tracking the attention of others, substantially increase children's tendencies to imitate across multiple domains, including artefact use and food preferences [22]. For example, observing an attention cue within the domain of ‘artefact use’ increased the likelihood of imitating the model's use of a different artefact by 13 times while increasing the copying of their food or drink choices by four times. Such work also reveals that children watch their models for cues of confidence, and deploy these in multiple domains [49,50]. Among adults, a long history of experimental work shows how information about a model's expertise in one domain influences their persuasiveness in other unrelated domains (see reviews in [4,46]), and recent work indicates that adults, like children, also use cues of confidence or pride displays [51] to target their cultural learning. In the fieldwork just discussed, cultural transmission networks reveal that Yasawans' perceptions of a model's success in one domain influences their willingness to learn from that model in other domains [47]. For example, perceiving someone as the best yam grower increases people's willingness to learn from them about yams by seven times, but similarly increases people's willingness to learn from these individuals about fishing and medicinal plants by between two and three times, even after controlling for learners' perceptions of their success or knowledge of fishing and medicinal plants as well as many other factors like age.

In the modern world, the power of celebrity endorsements (e.g. Beyoncé loves Pepsi) and in people's tendency to copy suicidal actions, including specific killing methods, from particularly prestigious individuals attests to the broad power of prestige-biased transmission [21]. In one recent well-studied case, the celebrity actor–director Angelina Jolie—who is neither a physician nor a medical researcher—wrote a *New York Times* OP-ED about her decision to get a double mastectomy after finding out that she had a genetic variant associated with an increased risk for breast cancer. Angelina's OP-ED initiated a flood of thousands of women seeking genetic screenings for breast cancer at clinics and on helplines in the UK, USA, Australia, New Zealand and Canada [52,53]. This flood continued for over six months.

Finally, much evidence indicates that humans use cultural learning to acquire costly social behaviours. In the laboratory, opportunities to observe prosocial models increase (i) *n*-person cooperation [54–57], (ii) altruistic giving (the extensive literature reviewed in [58, ch. 2]) and (iii) the punishment of inequitable offers [59]. In field experiments, cultural learning opportunities increase people's willingness to (i) help stranded motorists [60], (ii) volunteer [61], (iii) give blood, (iv) not jaywalk [62] and (v) donate to charity [63]. In both children and adults, these cultural learning effects are often large, and emerge in both naturalistic anonymous settings and one-shot economic games as well as in repeated economic games. The effects of cultural learning on one-shot altruism in anonymous contexts have also been shown to endure for weeks after exposure—so they are ‘sticky’, at least sometimes.^2^

## 3. The model

To explore whether prestige can promote the evolution of cooperation, we constructed a culture–gene coevolutionary model. We assume an infinite population in which a small fraction of the population are high status, and thus capable of pursuing leadership opportunities, such as hunting a turtle, cutting a canoe or leading a raid on another group. The remainder are low status, and thus potential *followers*. They may step forward and seize the reins of leadership, but if they do, no one follows them, so nothing happens. Individuals undergo the following life cycle:

(1) *Birth*. A generation is born with genetic traits that can potentially influence their social behaviour.
(2) *Childhood cultural learning*. Individuals culturally acquire a context-specific social behaviour (*x*) favouring either cooperation (e.g. *x* = 1, ‘always pitch in on the turtle hunt’) or defection (*x* = 0, ‘never help on the turtle hunt’) by copying a member of the prior generation with a probability proportional to their payoffs. This means that only cultural traits that raise an individual's payoff in the long run (in expectation) will proliferate. The frequency of cooperators (those with *x* = 1) after childhood cultural learning is *q*.
(3) *Social interaction*. Followers are randomly recruited into teams or groups of size *n* (*n* ≥ 2). Think of these as raiding parties, hunting teams or work groups. These groups are organized by a single leader who can be either cooperative or uncooperative based on her childhood learning (the *x*-value they acquired in Step 2).
(4) *Leader action and observation*. Group leaders either cooperate or defect based on the cultural trait they acquired during childhood. Followers observe their leader's behaviour in the social dilemma. Cooperative leaders pay a cost, *c*, to deliver a benefit, *b/n*, to each individual in their group.
(5) *Follower action*. Followers decide whether to cooperate or defect. This decision is based on their own *x*-value (based on their childhood enculturation) and on the probability, *p*, that they imitate their high status leader. One way to conceptualize this is that followers may be unsure whether their current context fits the context specified by their *x*-value. So, as both predicted by theory and demonstrated in much empirical work, followers may rely on cultural learning under uncertainty, especially when a particularly successful or prestigious model is readily available [58,64,65]. In the baseline model, we assume that copying the leader creates a permanent change in followers' *x*-values. However, we subsequently examine what happens if the effects of following the leader do not persist.
(6) *Payoffs*. All participants receive payoffs based on their own actions and those of others in their group according to a linear public goods game: the contributions made by all participants, including the leader, are summed and divided equally among all *n* participants regardless of whether they paid the costs (*c*) of cooperation.
(7) *Genetic and cultural reproduction*. This generation produces offspring in proportion to their payoffs (Step 1, above), and then acts as cultural models for the next generation (Step 2).

Existing work has revealed that prestige and leadership are complex, multifaceted phenomena. This mathematical model seeks to abstract away all that complexity and gain insight about just one unintuitive but potentially important dynamic: is the mere existence of prestigious individuals, acting as leaders, sufficient to catalyse a cascade of evolutionary pressures that cause societies to become more cooperative and prestigious individuals to be more generous? Intuitively, it is not obvious why followers would ever pay personal costs to blindly mimic a leader when they could benefit by defecting. Our model illuminates how, even in the absence of punishment, coordination benefits, efficiency or opportunity differences, or any other individual-level motivations to cooperate, the intragenerational dynamics of cultural learning can still cause societies to become steadily more cooperative once prestigious leaders exist. Consequently, in our model, groups are randomly composed every generation and interactions are one-shot (though leaders go first, and followers can then copy), to intentionally remove all effects of repeated interactions, genetic relatedness by common descent and intergroup competition. Leaders in our model have no special role in coordination, monitoring and sanctioning others’ behaviour, which allows us to isolate the effects of prestige-biased cultural learning alone on cooperation.

### (a) The baseline model: cultural evolution only

Our first step is to develop a baseline model for the cultural evolution of cooperation, which assumes all genetic traits are fixed. For convenience, we define the *net cost* (*C*) to an actor as: *C* = *c* − *b*/*n*, where *c* is the cost of cooperative action (as explained above) while *b*/*n* is the private benefit the actor gets back from his or her own action regardless of what others do. Under these assumptions, the cooperative behaviour (*x* = 1) will spread to fixation (*q* → 1) and remain stable when (see the electronic supplementary material for the derivation):3.1
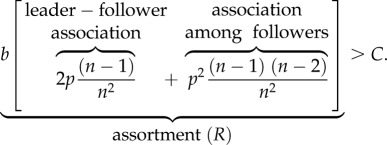


The bracketed term in (3.1) is measure of the phenotypic assortment generated by our model among the members of our *n*-person groups [66–68]. The large bracketed term *R* is composed of two parts that represent two different phenotypic relationships weighted by their relative contributions. The first term in *R* captures the association between leaders and followers created by the tendency of followers to copy their leader's behaviour. The second term, which involves *p*^2^, is the relationship between followers within a group created by the tendency of each follower to acquire behaviours from their leader. The term *p*^2^ is the probability that in any randomly selected pair both individuals copied the leader.

To see the importance of the prestige-bias and how it creates assortment, consider what happens when *p* approaches 0. In this case, we get *C* < 0, which is ruled out by assumption as uninteresting. Thus, cooperation will not evolve culturally unless *p* > 0. The bigger *p* is, the wider the range of conditions favouring cooperation.

Now, let us consider what happens in (3.1) when *n* is sufficiently large that we can assume *n* ≈ *n* − 1 ≈ *n*−2 and 1/*n* ≈ 0. With this assumption (3.1) becomes (3.2):3.2
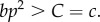


This makes sense. As the group expands, the leader becomes merely one among many, so her direct contribution to *R* is negligible compared to the associations she produces among her followers. Here, *R* reduces to just *p*^2^—the relationship between any two followers created by the fact that they both copied the leader. If followers copy their leader 50% of the time, *R* = 0.25—analogous to a group of half-brothers. If followers copy their leader a bit over 70% of the time, *R* = 0.5—a band of cultural siblings.

**Figure 1.**
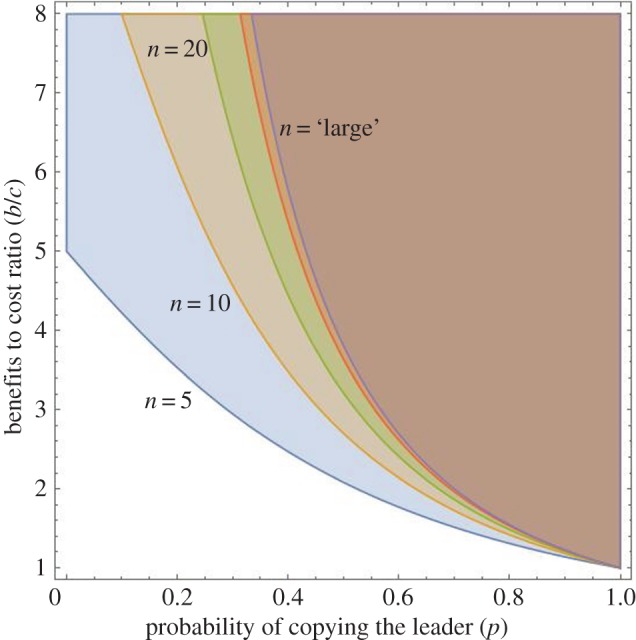
Conditions for the spread of a cooperative cultural trait. The figure plots the regions specified by equation (3.1) for *n* = 5, 10, 20, 100 and ‘large’.

Figure 1 illustrates how the group size, *n*, and the prestige effect, *p*, interact. The area above each curve shows the region of stable cooperation for five different group sizes (*n* = 5, 10, 20, 100 and ‘large’). First, note that *n* matters a lot when the prestige effect is weak (i.e. *p* is small). For example, when *p* is less than about 0.40, increasing *n* from 5 to 20 substantially reduces the region favourable to cooperation. And below about *p* = 0.20, cooperation is only viable in groups with less than about five individuals, and then only when cooperation really pays (high *b*/*c*). However, at the other end, when prestige has a big effect on followers (*p* is large), the size of the groups makes little difference and cooperation spreads under a wide range of conditions. In fact, for groups with more than 100 individuals, our ‘large’ approximation (3.2) provides the expected good fit. When *p* is greater than about 0.80, for example, groups with five individuals are not much more conducive to cooperation than much larger groups (for *p* > 0.80 look at the *b*/*c*'s favouring cooperation for *n* = 5 and *n* = ‘large’).

Of course, it is plausible that *p* and *n* are linked such that *p* necessarily declines as *n* increases. However, this may not always be the case, as some evidence suggests that humans use the attention of others as a ‘prestige cue’ [22], so seeing many others attending to someone may actually *increase* the model's transmission potential. Does the size of the global population necessarily diminish Angelina Jolie's prestige effects? This is one reason why we did not make *p* a function of *N*. We return to this issue in the discussion.

### (b) What if acquired cultural traits do not ‘stick’?

The Baseline Model above assumes that when followers copy their leaders these acquired traits ‘stick’, and can be passed on to the next generation via payoff-biased cultural transmission. However, such prestige effects may be ephemeral, as individuals gradually revert back to the ‘deeper’ traits they internalized as children. Or, alternatively, some fraction of the prestige effect (*p*) may be merely an act of deference to a high status individual (e.g. out of fear), and not represent the influence of cultural transmission. To address this, we now consider what happens when some of those who copied their leader ‘in the moment’ subsequently forget or lose what they acquired from the leader. That is, the follower copies either cooperation or defection from their leader for their action in the moment, but they later revert back to what they learned growing up, and pass this trait onto the next generation (in proportion to their payoffs). To formalize this, we assume that the traits acquired from leaders only endure (or ‘stick’) with probability *s*. This applies equally to both cooperation and defection.

Adding this to the Baseline Model, the condition for the spread of a cooperative trait via cultural evolution becomes3.3
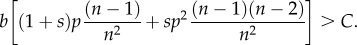


This is similar to (3.1): if *s* = 1, we get immediately back to (3.1). At the other extreme, if *s* = 0—nothing sticks and the prestige effect causes no intergenerational transmission—we obtain3.4
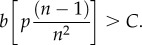


This inequality reveals a dramatic constriction of the conditions favourable to cooperation and is highly sensitive to *n* (declining as 1/*n*). If *n* is ‘large’, (3.4) is never satisfied. This shows that intergenerational transmission is crucial for the evolution of cooperation, especially for cooperation in groups larger than a few individuals. This also means that deference to high status individuals, whether it is derived from prestige or dominance (coercion), is the minor player in these models.

Now, letting *s* increase from zero, we can examine the effect of sticky prestige-biased cultural transmission. But, before turning to the plots, let us examine inequality (3.3) when *n* is large:3.5
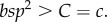


Here, *R* = *sp*^2^, which captures the long-run phenotypic assortment among followers created by sticky prestige-biased cultural transmission. If *p* = 0.7 and *s* = 0.5, *R* is about the same as among half-siblings (*R* = 0.25).

**Figure 2.**
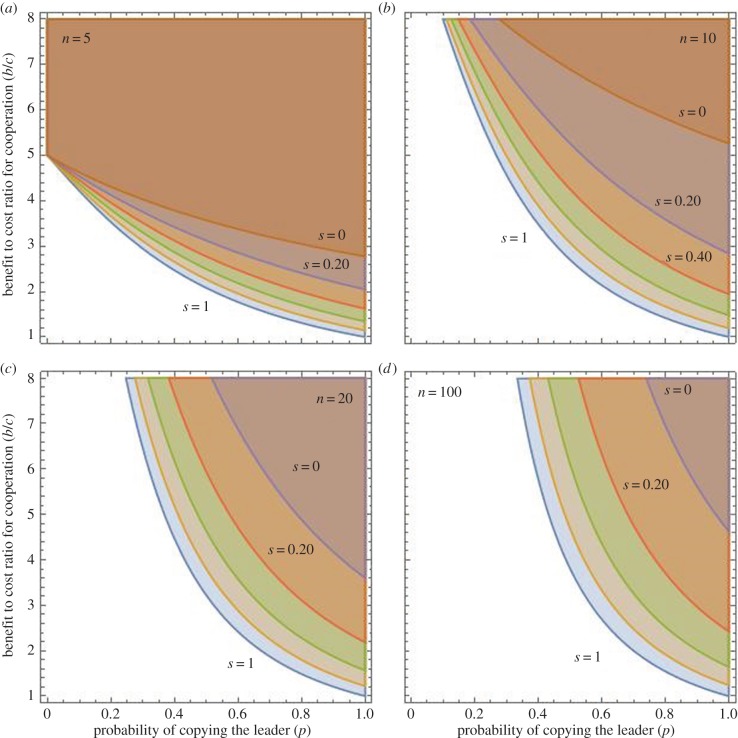
The effect of stickiness (*s*) on the conditions for the spread of a cooperative trait. (*a*) *n* = 5, (*b*) *n* = 10, (*c*) *n* = 20 and (*d*) *n* = 100. The curves in each subplot are for *s* = 0, 0.2, 0.4, 0.6, 0.8 and 1.

Figure 2 shows four different panels for (*a*) *n* = 5, (*b*) *n* = 10, (*c*) *n* = 20 and (*d*) *n* = 100. The curves for *s* = 0, 0.2, 0.4, 0.6, 0.8 and 1 on each panel carve out the region favourable to the spread of the cooperative trait. Together, the plots show that the stickier prestige-biased transmission is (the bigger *s* is) the broader the conditions favouring cooperation. However, in small groups with relatively low *p*-values, *s* has little impact on the conditions favourable to cooperation. By contrast, when *n* or *p* are large, increasing *s* substantially expands the range of favourable conditions.

### (c) Will a genetic variant that makes leaders more cooperative spread?

As we have shown, cooperation can evolve culturally because of how prestige effects create correlated phenotypes. This pattern opens the door for natural selection, operating in the wake of cultural evolution, to spread genes that make leaders more likely to adopt or express cooperative traits. Such a genetic variant spreads because by cooperating, prestigious leaders can cause their groups to become more cooperative—and they get an equal share of those induced benefits. Thus, we can now ask: under what conditions, if any, could such culture-driven genetic evolution occur?

We start with our Baseline Model (*s* = 1) and examine the conditions under which a genetic mutation could spread that makes leaders more likely to express a cooperative cultural trait over an uncooperative trait. That is, if a typical leader expresses the cooperative cultural trait with probability *Q*, when will natural selection favour a genetic variant that causes leaders possessing it to express with probability *Q* + *δ*. We begin by assuming this variant only expresses itself in leaders. Under these assumptions, more cooperative genetic variants will spread when3.6
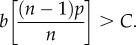


The bracketed term in (3.6) captures the extra benefits gained by a cooperative leader via the prestige effect on followers. If *n* is large (more than 50), this expression reduces to *bp* > *C*. Note that this condition is less strict than those derived above for the cultural evolution of cooperation (3.1). So, in this situation, if cooperation evolves culturally, then genes favouring more cooperativeness in prestigious leaders will always be favoured.

However, it is plausible that such a cooperative genetic variant might also sometimes ‘mistakenly’ be expressed in followers, causing them to cooperate more. To tackle this, let us assume that there is a genetic variant that always makes leaders more cooperative but makes followers more cooperative with probability *α*. Then, the condition for the spread of a cooperation-inducing mutation is3.7
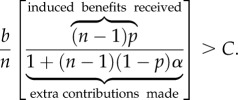


The term in the brackets is the ratio of the extra benefits that a more cooperative leader acquires from his followers (due to cooperation + prestige effects) to the extra costs paid by followers who ‘mistakenly’ contribute (these are the ‘bleed over’ costs of the mutant gene). Note that if *α* = 0, we return to (3.6), and if *n* is large, the condition is never satisfied.

**Figure 3.**
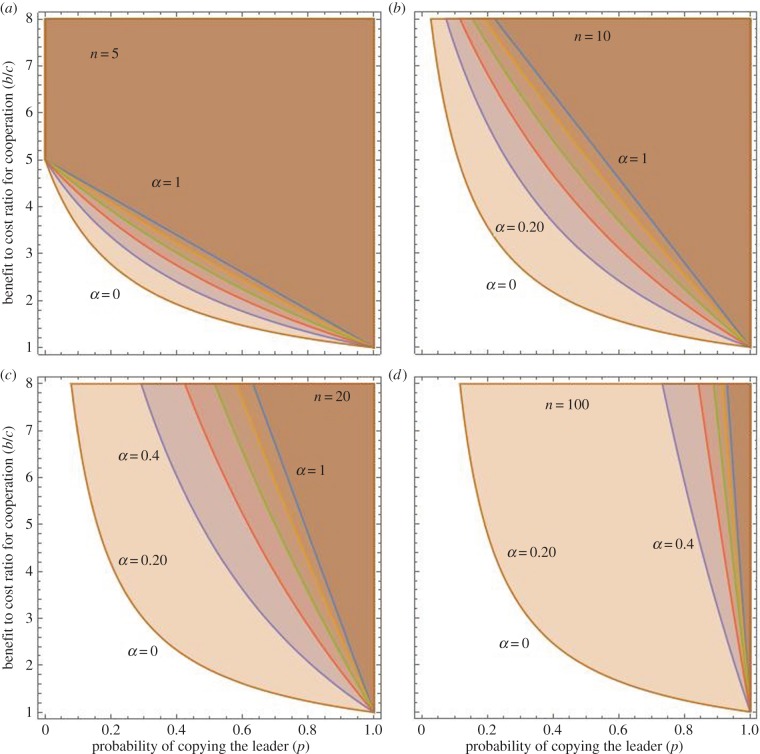
The conditions for the spread of genetic variants that promote cooperation among prestigious leaders. Each panel shows the curves for *α* = 0, 0.2, 0.4, 0.6, 0.8 and 1. (*a*) *n* = 5, (*b*) *n* = 10, (*c*) *n* = 20 and (*d*) *n* = 100.

Illustrating (3.7), figure 3 shows the conditions for the spread of a genetic variant that promotes cooperation among prestigious leaders. Each panel shows the curves for *α* = 0, 0.2, 0.4, 0.6, 0.8 and 1. The area above those curves is the region in which the cooperative mutation will spread. Each panel depicts a different value of *n*: (*a*) *n* = 5, (*b*) *n* = 10, (*c*) *n* = 20 and (*d*) *n* = 100. Perhaps the most important insight from this is that in small groups the ‘bleed over’ effect is relatively reduced compared with large groups. When *n* = 5, for example, *α* has relatively little effect, especially when *p* is either large or small. And, even when *α* = 1, there are ample conditions favouring the spread of a cooperative genetic variant (making both followers and leaders become more cooperative). By contrast, when *n* = 100, even a 20% chance of a ‘mistaken’ expression in followers dramatically shrinks the favourable conditions. The effects of *α* are already evident when *n* = 20.

Inequality (3.7) and figure 3 suggest an interesting psychological prediction: prestigious leaders should be relatively more cooperative in small groups (*n* = 5) but not in large groups (*n* = 100). That is, cooperation-enhancing genetic variants that facultatively express only in small groups will be favoured. The intuition here is that in large groups many mutant followers suffer the costs of cooperation while only one leader benefits from his or her cooperative action. Meanwhile, in small groups, relatively fewer followers suffer.

Finally, we framed this as being about a genetic variant. However, it could also be thought of as a cultural trait, such as a story script, that is acquired early, and evolves more slowly.

### (d) Will selection favour reducing *p*, the prestige effect

In developing these ideas, we assumed that learners were constrained from figuring out whether various elements in their model's behavioural repertoire were causally connected to their success or prestige. That is, to some degree (captured by our *p* parameter), individuals have to copy prestigious individuals across many domains, including in the social dilemma used in our model. If they do not copy broadly, we assume they will miss out on learning some important fitness-enhancing traits. Thus, we have constrained natural selection from sharpening learners abilities to accurately pick out only the fitness-enhancing traits possessed by their models. While this assumption is plausible [21], it is nevertheless worth relaxing this constraint to see if selection in our model will favour reducing *p*, or even drive it to zero. To study this, we take our Baseline Model and ask whether genetic mutants with smaller *p*-values can invade the cooperative equilibrium. Note we make the conservative assumption that our mutants can do this without fitness penalties for retasking existing brain tissue or for inefficiencies introduced into their learning in other domains.

The result is simple. Mutants with lower *p* values are not favoured by natural selection. Instead, such genetic variants are selectively neutral. To see why, realize that at the culturally evolved cooperative equilibrium, cooperation is favoured and common. Mutants will tend to already have the cooperative cultural trait, having acquired it via payoff-biased cultural learning during childhood. Thus, a rare mutant gets neither an advantage at the cooperative equilibrium from not copying the leader nor a disadvantage.

Supporting our initial assumption, this result implies that any exogenous constraint, even a weak one, that imposes a cost on distinguishing our key social dilemma from all the other fitness-relevant domains—in which one would benefit from relying on cultural learning—will prevent the invasion of mutants who refuse to copy the leader (the deterioration of *p*).

## 4. Discussion

Motivated by empirical patterns of leadership observed across diverse societies and by recent work on the evolution of prestige, we have developed a set of culture–gene coevolutionary models that explore the conditions under which the existence of prestige-biased cultural transmission can favour both the cultural evolution of cooperation and the genetic evolution of prosocial proclivities in prestigious leaders. Rooted in informational asymmetries among individuals, these models allow us to begin to draw novel connections between the evolution of prestige, cooperation, prosocial motivations and leadership, and provide a firmer foundation for making predictions about behaviour and psychology. In this final section, we (i) highlight key insights and empirical predictions derived from our models, (ii) discuss recent empirical work that provides preliminary evidence for our predictions, and (iii) outline the weaknesses of our models and highlight key directions for future work.

### (a) Summary

We derive four key insights and various predictions from our models.

(1) Prestige-biased transmission can favour the evolution of cooperative cultural traits by creating phenotypic associations, both between leaders and followers, and between followers. As groups expand, our phenotypic association, *R,* approaches *p*^2^, which is the probability that any pair of individuals share the same cultural trait due to transmission from the leader. This means our mechanism operates by assortment, the general process underlying many models of cooperation including those based on kinship, reciprocity and signalling [58,69–72]. Our model provides two specific empirical predictions: (i) individuals with larger prestige effects (*p*) will be able to sustain more costly cooperation in larger groups—inequality (3.1); and (ii) increasing group size makes it harder to sustain cooperation, though this effect is muted in already large groups or with very prestigious leaders. All these effects are nonlinear.
(2) The emergence of cooperation depends heavily on the tendency of followers to retain the behaviours they acquire from leaders (*s*) and pass those on in the future. If followers merely go along with their leader, say out of deference, fear or in hope of reciprocal benefits, the conditions favourable to cooperation shrink dramatically. This may explain why high status individuals in non-human primate groups cannot generate much cooperation—primate studies reveal little, if any, enduring prestige-biased cultural transmission [73]. Thus, if it turned out that behaviours acquired by humans via prestige-biased transmission were merely ephemeral, then our model would predict little cooperation and would be unable to account for the nexus of prestige, cooperation and leadership observed empirically.
(3) Natural selection operating on genes will often respond to these culturally evolved cooperative patterns by favouring genetic variants that make leaders more prosocial—more likely to behave cooperatively. However, this is only true for smaller groups because too many followers suffer in larger groups. This suggests two empirical predictions: (i) individuals with larger prestige effects (*p*) will tend to be more prosocial, but (ii) these prosocial inclinations will evaporate in larger groups. It is important to realize that these predictions do not contradict the above predictions (in 1) regarding cooperation in large groups. Here, the idea is that leaders will have an *even greater tendency* towards prosociality than they would otherwise. That is, when they find themselves in small groups, particularly prestigious leaders will more strongly adhere to their culturally acquired cooperative norms relative to their baseline tendencies to stick to such norms (because there are additional evolutionary incentives).
(4) Natural selection does not favour reducing the prestige effect (*p*) under the conditions created by cultural evolution. Any small external constraint will prevent an invasion by individuals with lower *p* values. This predicts that prestige-biases will still operate in social dilemmas (as seen below).

In the light of these results, it is worth considering how cultural evolution might have amplified, or otherwise harnessed, this cooperation-inducing mechanism. For example, *n* and *p* may be linked in some way, such that *p* tends to decline as *n* increases. However, institutions, norms and technologies may mitigate this effect, or even reverse it. In particular, individuals seeing a large crowd attend to and respond to a skilled orator or renowned leader may be powerfully affected—raising their *p* value for that leader. Though the size of a crowd that one person can speak to is limited, without big screens, well-designed acoustics and powerful sound systems, some groups may have figured out ways around this. The Plains Indians, for example, engaged in oratory in very large ceremonies using a gestural sign language that involved expansive movements that were visible at a distance [21]. Similarly, writing, radio and television may permit one leader to sustain or increase his average *p* value even in a large group, as might the winning of democratic elections.

It is also worth considering whether an oral tradition might gradually increase the *p* value of a prestigious leader, perhaps even after his or her death. In the absence of the leaders themselves, stories of their heroic acts may spread far and wide, and inspire the young to set higher standards for themselves, and to mimic the valour and sacrifice of their heroes. Ethnographic evidence suggests that particularly prestigious Big Men gradually transformed after their deaths into even more powerful ancestor spirits, as the repeated retelling of their stories magnified their talents, successes and even their physical size [74].

Thus, it is plausible that groups may vary in how effectively their institutions and beliefs harness the Big Man Mechanism. Fuelled by such between-group variation, intergroup competition may drive cultural evolution to favour those groups or institutional forms that most effectively exploit this cooperation-enhancing mechanism.

Overall, our effort has been to focus a narrow theoretical beam on one, heretofore unanalysed, aspect that may be important for understanding the nexus of prestige, leadership and cooperation. Of course, as we have emphasized, many other factors and mechanisms no doubt influence both the cooperation generated by leaders and the tendencies of leaders themselves towards prosociality. Our approach, however, makes several unique predictions, just outlined, that could be addressed through a combination of experimental and observational approaches (see below for laboratory experiments), including natural field experiments. One implication of our approach is that our prestige-cooperation effects should be limited to social species with sufficiently high levels of cultural transmission. This arguably eliminates most animals, and all non-human primates [73], though it may not eliminate elephants or cetaceans [21, ch. 8]. Nevertheless, in contrast to our model, other approaches such as those based on reputation, kin-based allies, signalling and competitive altruism should all readily apply to non-human primates, and predict high levels of leader-based cooperation. To our knowledge, no evidence supports these predictions for non-human primates. Thus, we suspect our mechanism may lay a human unique, or nearly unique, foundation on which these other cooperation-generating mechanisms can further build.

### (b) Existing experimental evidence

Existing evidence from laboratory experiments on ‘first-movers' [75] supports the general link among prestige, cultural learning and cooperation, and the prediction that more prestigious individuals will tend to become more prosocial when permitted to take the lead [76–81]. In one experiment [82], players participated in a trivia contest prior to playing a series of sequential Prisoner's Dilemmas. The trivia contest provided an opportunity to endow some individuals with gold stars, congratulatory ribbons and applause (a minor prestige boost) while leaving others unadorned. Though players assumed that the gold stars, etc., reflected performance in the contest, they actually derived from an arbitrary feature of what the players wrote on their contest forms—so, players were randomly assigned to the high and low prestige treatments. After the trivia contest, pairs of players then repeatedly engaged in a series of one-shot, anonymous, sequential Prisoner's Dilemmas. Half of the time the gold-starred individuals went first, and could either cooperate or defect first, and half of the time they went second. All pairing involved one high prestige (gold-starred) player and one low prestige (non-starred) player. Despite the relative weakness of this prestige manipulation, when high prestige players went first, they were copied by low prestige players 45% of the time, while when low-prestige players went first they were only copied by high prestige players 30% of the time. Getting the gold star also made individuals more likely to cooperate, but only when they went first. High prestige players cooperated 55% of the time when they went first, whereas low prestige players cooperated only 33% of the time when they went first. By contrast, when the high prestige players went second, they cooperated only 13% of the time (less than low prestige players going either first or second). These behavioural differences cashed out into big payoff differences in the aggregate: pairs in which the high prestige player went first earned 80% more money than did pairs in which the low prestige player went first.

Such effects seem well known to charitable organizations and universities who begin their fund-raising campaigns by allowing particularly prestigious individuals to take the lead, and make large contributions. When asked why the university requests permission from big donors to announce their contributions, the chairman of Johns Hopkins trustees explained, ‘fundamentally we are all followers. If I can get somebody to be a leader, others will follow. I can leverage that gift many times over’ [82].

Our modelling approach contributes to this empirical literature in several ways. First, we provide an ultimate-level explanation for why first movers are so powerful in elevating cooperation even in one-shot anonymous experiments in which neither reputation nor competitive giving can operate. It is unclear how other approaches to leadership explain these empirical patterns.^3^ Second, our model provides an explanation for why something like a trivia contest, which bears no resemblance to a social dilemma, could influence cooperation. Finally, our overall framework explains why some individuals might be inclined to move first and cooperate, because they will have more optimistic beliefs about how cooperative the world will be if they—and not others—go first [75].

### (c) Weaknesses and ongoing work

Like all formalisms in evolutionary biology, our models abstract from the real world in an effort to illuminate a particular set of processes. Future modelling work should examine the effects of finite populations, intergroup competition, repeated interactions within groups and continuous cultural traits (instead of our dichotomous ‘cooperate’ or ‘defect’) as well as the impact of other well-established forms of cultural learning, such as conformist transmission [83,84] or credibility enhancing displays [85]. In our view, the most important elements missing from the models above involve (i) competition among aspiring leaders within a single group, (ii) the ability of more prestigious or cooperative leaders to recruit relatively larger groups of followers, and (iii) the addition of cultural traits involving costly punishment. Competition among aspiring leaders might, for example, elevate contributions to entice more followers, when followers face a choice [32]. Or a tendency to punish non-cooperators may spread among followers just like cooperation does, boosting cooperation even further. In future work, we will present detailed models of these dynamics.

## Supplementary Material

Supplementary material

## Endnotes

1 Though not the place to substantially critique existing lines of research, we note that several models of leadership and the evolution of cooperation based on reputation (including competitive altruism), signalling and punishment have largely not addressed the equilibrium selection problem [33]. Hooper *et al.*'s model [29], for example, generates many stable equilibria, but the authors do not explain how or why the cooperative-leadership equilibrium is selected. Notably, these models could work well as cultural evolutionary models, where intergroup competition can select among stable equilibria, but this is unlikely to be important for genetic evolution [34,35].

2 Such cultural learning effects, even in cooperative dilemmas with the potential for free-riding, are less surprising once one realizes that real-world situations, unlike most economic games or models, do not come categorized (e.g. ‘coordination game’) or labelled with payoffs [34]. Young foragers, for example, who grow up in a society with food taboos that foster sharing [21] often would not know what would actually happen if someone violated the taboos—people do not have any idea what the off-equilibrium payoffs are. Will he be punished or poisoned? Will the punishment be natural, a beating or supernatural, an illness or bad luck in hunting? Whether this is actually an *n*-person cooperative dilemma with free-riding opportunities depends on the answers to such difficult questions. Such uncertain social situations are not unlike the many non-social situations we face, and are precisely the circumstances that cultural learning evolved to help us navigate [20,58,64].

3 Explanations based on a misfiring of innate psychological mechanisms that evolved in a world supposedly devoid of low-frequency interactions, secrets or anonymity face substantial theoretical and empirical challenges [33].
