# Supplementary material for: The Big Man Mechanism: how prestige fosters cooperation and creates prosocial leaders
Source: Philos Trans R Soc Lond B Biol Sci. 2015 Dec 5;370(1683):20150013. doi: 10.1098/rstb.2015.0013 (PMC4633849; doi:10.1098/rstb.2015.0013)
Supplement: Supplementary material [file rstb20150013supp1.pdf]

doi: 10.1098/rstb.2015.0013

Supplemental for *The Big Man Mechanism*

Joseph Henrich, Maciej Chudek, Robert Boyd

Philosophical Transactions of the Royal Society B

Overview

Here we develop a simple model of the influence of prestigious leaders—individuals whom others copy—on the spread of a cooperative trait. Our model is designed to explore the tension between two competing influences on the long term frequency of cooperative traits—influential leaders are imitated, but so are fitter individuals (with higher payoffs) and these influences may conflict.

Individuals in the population go through a two stage life-cycle: During the first stage, children acquire genetic and cultural traits in proportion to the fitness or payoff of the individuals in the previous generation. At this stage, the frequency of the cooperative cultural trait is q.

During the second stage, individuals form into groups where they may choose to cooperate. Each group is is composed of n individuals and has only one leader, who acts first to cooperate or defect according the traits he or she acquired in the first stage. Cooperation is modelled as a one-shot public goods interaction. Cooperators increase their group-members' fitness at a cost to themselves. At this stage, followers copy the actions of their leader with probability p, so a fraction p (on-average) of the followers abandon their childhood cultural trait, either temporarily or permanently, and instead imitate the behaviour performed by the leader. Followers keep the trait they acquired from their leader to potentially pass on to the next generation with probability s (initially, we assume $s = 1$). Here we need to keep track of just how many individuals have switched, in which kinds of groups, and what their fitnesses are. We do so by using sub- and super-scripted X variables, which we define below.

To be clear, leaders in our model do not compete or pay costs to become leaders; they do not vary in their ability to attract or sway followers, though obviously they are different from followers (who cannot attract followers); they have no special ability to compel or reward followers (though such an ability could be assumed to be part of p); and, they do not receive any exogenous fitness advantage.

Finally, we tally everyone's payoffs across all the kinds of groups and interactions (using sub- and super-scripted V variables), and are ready to see its impact on the frequency of the cooperative trait (q) in the next generation.

We extend the model by asking about the relative fitness of mutants who are slightly more or less inclined to cooperate or imitate leaders than the average individual.

For brevity, here in the supplemental we use "Good" to mean "leaders who cooperate" and/or "groups with leader who cooperates", and Bad to mean "leaders who do not cooperate" or "groups with leader who do not cooperate".

Definitions

Types

These are the kinds of individuals and groups in the world. We use upper case to designate cooperative types.

Set	Description
$\mathbb{L} = \{L, l\}$	There are two kinds of *Leaders*, those who cooperate (L) and those who don't (l).
$\mathbb{F} = \{F, f\}$	There are two kinds of *Followers*, those who cooperate (F) and those who don't (f).
$\mathbb{I} = \{\mathbb{L}, \mathbb{F}\}$	There are two kinds of individuals: leaders and followers.
$\mathbb{J} = \{G, g\}$	There are two kinds of groups, those with leaders who cooperate (G), and those with leaders who don't (g).

Parameters and Variables

Parameter	Description
$n \geq 2$	Number of individuals (including leader) in a group
$b > c$	Group benefit of each cooperative act
$c > 0$	Individual cost of each cooperative act
$C = c - \frac{b}{n}$	Individual net cost of each cooperative act
$s \in [0, 1]$	Probability that the behaviour copied from a leader *sticks* (i.e., is transmitted to the next generation by a follower).
$p \in [0, 1]$	The proportion of individuals who imitate a *Leader*
$Q \in [0, 1]$	The proportion of *Leaders* disposed to cooperate
$q \in [0, 1]$	The proportion of *Followers* disposed to cooperate
$\delta_x \in [-x, 1 - x]$	A mutation in some variable (x).
$\alpha \in [0, 1]$	The probability that a genetic disposition to be more cooperative as a leader spills over when the gene expresses in a follower.

Note that in most of our models, $Q = q$. However we will express leaders' and followers dispositions to cooperate separately to more easily extend our model model to explore the pressures on a cooperative gene that expresses differently in leaders and followers, below.

Frequencies

After imitation, we can express the frequency of each type in each group ($X_{i \in \mathbb{I}}^{j \in \mathbb{J}}$) as:

Leaders

$$
\begin{aligned}
X_L^g &= 0 \\
X_l^G &= 0 \\
X_L^G &= \tfrac{1}{n}Q \\
X_l^g &= \tfrac{1}{n}(1-Q)
\end{aligned}
$$

Followers

$$
\begin{aligned}
X_F^G &= Q\tfrac{n-1}{n}(q+(1-q)ps) \\
X_f^G &= Q\tfrac{n-1}{n}(1-q)((1-p)+p(1-s)) \\
X_F^g &= (1-Q)\tfrac{n-1}{n}q((1-p)+p(1-s)) \\
X_f^g &= (1-Q)\tfrac{n-1}{n}((1-q)+qps)
\end{aligned}
$$

Payoffs

Within a group, a focal individual receives benefits in proportion to how many other non-leaders (i) cooperate. Here we simplify later expressions by expressing these benefits for Good (Z^G) and Bad (Z^g) groups respectively, including a baseline fitness term (w_0):

$$
\begin{aligned}
Z^G(i) &= w_0 + \tfrac{b}{n} + i\tfrac{b}{n}\,(q+(1-q)p) \\
Z^g(i) &= w_0 + i\tfrac{b}{n}(1-p)q
\end{aligned}
$$

These functions make it easy to express the payoff to each of the types ($V_{i\in\mathbb{I}}^{j\in\mathbb{J}}$):

Leaders

$$
\begin{aligned}
V_L^G &= Z^G(n-1) - c \\
V_l^g &= Z^g(n-1)
\end{aligned}
$$

Followers

$$
\begin{aligned}
V_F^G &= Z^G(n-2) + \tfrac{b}{n} - c \\
V_f^G &= Z^G(n-2) \\
V_F^g &= Z^g(n-2) + \tfrac{b}{n} - c \\
V_f^g &= Z^g(n-2)
\end{aligned}
$$

We know that the average fitness must be

$$
\bar{V} = \sum_{i\in\mathbb{I}}\sum_{j\in\mathbb{J}} X_i^j V_i^j
$$

The Baseline Model

For the Baseline Model we assume that $s = 1$.

When will a culturally-encoded trait to cooperate spread?

We know that q is the frequency of cooperators before imitation, X is their frequency after imitation, that V is their payoffs, and that the next generation spawns in proportion to payoffs. We can ask when the next generation will have more cooperators than the current by considering the conditions that satisfy:

$$\sum_{j\in\mathbb{J}} \left(X_L^j V_L^j + X_F^j V_F^j \right) / \bar{V} - q \quad > \quad 0$$

This is satisfied so long as:

$$n^2(1-q)q(w_0 + q(b-c)) \left(b(n + (n-1)p + (n-1)ps(1 + (n-2)p)) - cn^2 \right) > 0$$

Note that $n^2(1-q)q(w_0 + q(b-c)) \geq 0$, allowing us to hone in further on the difference that makes a difference:

$$\frac{b}{n}\left(1 + \frac{1}{n}(n-1)p + \frac{(n-1)p}{n}(1 + (n-2)p)\right) > c$$

or

$$\overbrace{C = c - \frac{b}{n}}^{\text{Coop. costs}} < \overbrace{\frac{b}{n}}^{\text{Benefits}} \left(\overbrace{\underbrace{\frac{1}{n}}_{\text{freq.}} \underbrace{(n-1)p}_{\text{donations}}}^{\text{to coop. leaders}} + \overbrace{\underbrace{\frac{(n-1)p}{n}}_{\text{freq.}} \underbrace{(1 + (n-2)p)}_{\text{donations}}}^{\text{and imitators of coop. leaders}} \right)$$

What if the prestige effect isn't sticky? Are deference and coercion enough?

In the previous model cooperation can spread for two reasons: because cooperators have higher payoffs (and so spawn more cultural progeny) and because people copy cooperative leaders. We can separate these two components by assuming $0 < s < 1$.

So, if followers are equally likely to forget behaviours learned from Good and Bad leaders, cooperation spreads when this condition is satisfied:

$$\frac{b}{n}\left(\frac{1}{n}(n-1)p + s\frac{(n-1)p}{n}(1+(n-2)p)\right) > c - \frac{b}{n} = C$$

That is, the benefits earned by cooperative followers are discounted by the stickiness rate.

Will a genetic variant general cooperativeness spread?

Above we tracked the spread of a culturally transmitted cooperative behaviour. We saw that there are conditions under which the existence of prestigious leaders can cause this trait to spread. Over long periods of time many such cooperative cultural innovations might arise and spread by this mechanism. While these cultural traits are spreading, individuals who adopt them sooner than others get an advantage when they are leaders because they have more cooperative followers sooner, or a disadvantage when they are followers, because they cooperate when it is individually disadvantageous.

Here we ask whether a trait (either genetically or culturally transmitted) that makes individuals more cooperative will be favoured by these same mechanisms. We allow for the possibility that the trait might be selectively expressed in Leaders (e.g., a gene might be activated by hormones more common in higher status individuals, or a script might be encoded as a story told to children about the behaviour of ancestral leaders). However selective expression may not be perfect and thus is sometimes expressed in followers too.

To formalize this idea, suppost that there are rare "mutants" who cooperate at a rate higher than the base rate of the cultural trait. That is, if a typical Leader in the population expresses the cultural trait with probability Q, the mutant does so with probability $Q + \delta_Q$, where $\delta_Q \in [0, 1-Q]$ (we do not need to assume that δ_Q is small because our results are monotonic in δ_Q anyway). When they are followers, a spillover effect causes the mutants to have a correlated increase in their chances of cooperating: $q + \alpha\delta_Q$. We ask these when mutants have higher viability (V_{δ_Q}) than average non-mutants ($\bar{V}$):

$$\begin{aligned}
V_{\delta_Q} &= \textstyle\sum_{j\in\mathbb{J}} \left(X_L^j V_L^j\right)|_{Q=Q+\delta_Q} + \sum_{j\in\mathbb{J}} \left(X_F^j V_F^j\right)|_{q=q+\alpha\delta_Q} \\
V_{\delta_q} &> \bar{V} \\
c &< \tfrac{b}{n}\left(1 + \tfrac{(n-1)p}{1+(n-1)(1-p)\alpha}\right)
\end{aligned}$$

$$\overbrace{C = c - \frac{b}{n}}^{\text{Coop. costs}} < \overbrace{\frac{b}{n}}^{\text{Benefits}} \underbrace{\frac{\overbrace{(n-1)p}^{\text{Extra contribution } (bs) \text{ received}}}{1 + (n-1)(1-p)\alpha}}_{\text{Extra contributions given}}$$

Thus, the existence of prestigious leaders can facilitate the spread of genes or cultural scripts that lead to a generalised increase in likelihood of cooperation. This is especially likely for genes or norms that express selectively in leaders (i.e., when α is small, making the RHS larger). It is also more likely to happen the more likely individuals are to imitate leaders (i.e, as p becomes large).

Note the simplifications for the two boundary cases. When the mutation occurs exclusively in leaders, it spreads when:

$$\frac{b}{n}(1 + p(n-1)) > c$$

When n is large, the LHS increases approximately linearly in p to $b > c$ when $p = 1$.

When the mutation is indiscriminant with respect to status, occurring equally in leaders and followers, it spreads when:

$$\frac{b}{n}\frac{1}{n - p(n-1)} > c$$

Note that this has a very different relationship with n. When n is large, the condition is very difficult to satisfy, unless p is very close to one.

Will a tendency not to imitate leaders spread?

There are several ways to ask this question. A simple one is to consider the fate of a mutant who imitates leaders at a higher or lower rate, $p + \delta_p$. Specifically:

$$\begin{aligned} V_{\delta_p} &= \textstyle\sum_{i \in \mathbb{I}} \sum_{j \in \mathbb{J}} (X_i^j V_i^j)\big|_{P=p=p+\delta_p} \\ V_{\delta_p} - \bar{V} &> 0 \end{aligned}$$

Here, we find that in this degenerate case: $V_{\delta_p} = \bar{V}$. Fitness doesn't depend on how likely you are to imitate. The benefits you gain by imitating defectors more are offset exactly by the costs of imitating cooperators. This means that p is set by external contraints, which is exactly the assumption we began with.
